# An overview of the sepsis situation in the Department of Infection Diseases, University Hospital Center, Tirana

**DOI:** 10.1186/cc11801

**Published:** 2012-11-14

**Authors:** E Puca, A Pilaca, P Pipero, J Gremi, P Luga, G Stroni, E Qyra, I Akshia, D Kraja, E Puca

**Affiliations:** 1 University Hospital Center 'Mother Teresa', Tirana, Albania; 2 American Hospital, Tirana, Albania

## Background

Sepsis is a condition that has been known since 2,700 years ago, but it is still a serious threat for human health [1]. Sepsis is a serious public health problem [2]. It is a complex process that can affect any individual and can originate from multiple sites and be caused by multiple microorganisms [3].

## Methods

We conducted a retrospective, observational study at University Hospital Center, Tirana, involving 535 critically ill patients diagnosed with sepsis admitted to the Department of Infection Diseases from January 2006 to December 2011. Blood cultures were performed on the admission day.

## Results

During the study period there were 535 patients with sepsis admitted to our hospital. From these, 288 (53.83%) were males and 247 (46.16%) were females. In the ICU, 103 cases (19.25%) with diagnosis of sepsis or severe sepsis were admitted. The urinary tract was the predominant site of infections in 39.81% of cases, followed by the lungs in 34.95% of cases, intra-abdominal infection (not postoperative) in 15.7% of cases, soft tissues in 2.24% of cases and with unknown origin in 7.28% of cases. Organ failure (severe sepsis or multiorgan dysfunctions) was found in 37.9% of patients. Blood culture results were positive in 23% of the patients. See Table 1.

**Table 1:** Distribution of cases with sepsis in department of infection diseases during 2006 to 2010

	2006	2007	2008	2009	2010	2011	Total
Cases	59	57	63	76	122	158	535
Males	34	35	27	41	69	82	288
Females	25	22	36	35	53	76	247
Mean age	44.28	47.59	44.46	40.56	48.88	49.92	45.94
Standard deviation	18.98	18.96	19.18	18.77	20.03	19.34	19.21

## Conclusion

Our study shows a progressive increase in the incidence of sepsis mostly in the last years. This could be explained by the possibility of increasing cases with sepsis or the doctors being affiliated with sepsis terminology. Urinary tract infection was the leading cause of sepsis. These data could be explained because our hospital is not the only center for treatment of sepsis and in our country we have another hospital for the treatment of lung diseases. The low value of positive culture results could be explained by our health system problems, using antibiotics much more than needed or antibiotic therapy being started before admission to our hospital. However, we propose that a developing country like Albania needs to develop a national center for sepsis.
